# Effects of de-escalated bisphosphonate therapy on bone turnover biomarkers in breast cancer patients with bone metastases

**DOI:** 10.1186/2193-1801-3-577

**Published:** 2014-10-01

**Authors:** Christina L Addison, Gregory R Pond, Huijun Zhao, Sasha Mazzarello, Lisa Vandermeer, Robyn Goldstein, Eitan Amir, Mark Clemons

**Affiliations:** Program for Cancer Therapeutics, Ottawa Hospital Research Institute, Ottawa, ON Canada; Department of Medicine, University of Ottawa, Ottawa, ON Canada; Biochemistry, Microbiology and Immunology, University of Ottawa, Ottawa, ON Canada; Department of Oncology, McMaster University, Hamilton, Canada; Princess Margaret Hospital, Toronto, ON Canada

**Keywords:** Bisphosphonate, Bone metastasis, Breast cancer, Biomarker, Skeletal related event, De-escalated therapy

## Abstract

While de-escalation of bisphosphonates from 4 to 12-weekly dosing has been shown to be clinically non-inferior to standard dosing, there is evidence the de-escalation is associated with increased bone turnover biomarkers. Here we evaluated the effect of de-escalated dosing on a panel of biomarkers and determined their association with incidence of skeletal related events (SREs) in breast cancer patients with ‘low risk’ bone metastases. As part of a pilot randomized trial, women with baseline C-telopeptide levels <600 ng/L after >3 months of 3–4 weekly pamidronate were randomized to continue pamidronate every 4 weeks or de-escalation to 12-weekly treatment. Serum was analysed for bone biomarkers (C-telopeptide, N-telopeptide, bone-specific alkaline phosphatase, transforming growth factor-β, procollagen type 1 N-propeptide, activinA and bone sialoprotein) using ELISA. The associations between changes in biomarkers, pain scores and SREs were assessed by univariable logistic regression. Numerical increases in all biomarkers were observed between baseline and 12 weeks but were of higher magnitude in the de-escalated arm. Pain scores in the de-escalated treatment arm showed a greater magnitude of pain reduction from baseline to 12 weeks. Neither baseline levels nor changes in biomarkers from baseline to 12 weeks on treatment were associated with on study SREs. Baseline pain as measured by the FACT-BP was associated with increased risk of SRE. In conclusion, biomarkers of bone activity do not appear to predict for SREs in ‘low risk’ cohorts. However, baseline bone pain appears to be associated with SRE occurrence, a finding which warrants evaluation in larger cohorts.

**Electronic supplementary material:**

The online version of this article (doi:10.1186/2193-1801-3-577) contains supplementary material, which is available to authorized users.

## Introduction

Bone is the most common site of metastasis in breast cancer (Coleman & Rubens 1987) and skeletal metastases are associated with skeletal related events (SREs) such as; surgery/radiation to bone, pathological fractures, spinal cord compression and hypercalcemia. Development of SREs can significantly impair a patient’s quality of life, and thus current treatment strategies attempt to prevent or delay the occurrence of SREs. As the osteoclast is the primary cell type implicated in bone destruction, osteoclast-inhibiting agents such as bisphosphonates or denosumab are essential components of the management of patients with bone metastases (Clemons et al. 2012; Coleman 2011; Dougall et al. 2014; Drooger et al. 2013).

Initial studies of bisphosphonates, treated patients every 3–4 weeks concurrently with chemotherapy. However, given the long terminal half life of these agents, and the fact that patients may be treated with them for years, their dosing frequency has come into question (Kimmel 2007), leading to a number of trials looking at de-escalated therapy. To date both single arm (Addison et al. 2014) and randomized trials (Amadori et al. 2013; Hortobagyi et al. 2014; Amir et al. 2013) have confirmed that de-escalated treatment is feasible and at least over the short term is not associated with a greater risk of SREs. It is also noteworthy that cumulative exposure to these drugs is associated with significant toxicities (Mariotti 2008), which could be reduced with use of de-escalated treatment strategies. One potential caveat to de-escalated regimens is that bone-turnover markers such as N-telopeptide (NTx) (Amadori et al. 2013) or C-telopeptide (CTx) (Amir et al. 2013) which are often used to monitor bone metastases progression appear to rise more substantially in de-escalated arms. These findings raise questions around the long term safety of de-escalation of bone targeted therapy.

The REFORM trial was a pilot randomized trial in which breast cancer patients with bone metastases considered to be at “low risk” of complications from their metastases (based on levels of the bone turnover marker CTx <600 ng/L) were randomized to continue therapy once every 3–4 weekly (standard) or to de-escalate to treatment once every 12-weekly for 48 weeks (Amir et al. 2013). The primary analysis comprised changes in serum CTx, however patients were also followed for the occurrence of SREs. While changes in CTx were not associated with SRE risk in the REFORM trial the utility of other putative biomarkers of bone activity as predictors of SREs remain of interest. With availability of longer follow-up data we now explore a series of bone biomarkers including CTx (Lipton et al. 2008; Lipton et al. 2011; Coleman et al. 2005), urinary (u)NTx (Rosen et al. 1994; Gorai et al. 1997; Clemens et al. 1997), bone-specific alkaline phosphatase (BSAP), transforming growth factor (TGF)-β (Baselga et al. 2008; Desruisseau et al. 2006), pro-collagen type 1 amino terminal peptide (P1NP) (Garnero et al. 2000a; Chevrel et al. 2007), activinA (Reis et al. 2002), bone sialoprotein (BSP) (Diel et al. 1999) and pain scores (Harris et al. 2007; Broom et al. 2009) and their association with the incidence of SREs in the REFORM trial.

## Materials and methods

### Patient cohorts

REFORM was a pilot, randomized, non-inferiority trial designed to explore the effect of de-escalated bisphosphonate therapy on women with breast cancer and bone metastases who were biochemically defined as being at low risk of SREs. Women with histologically proven breast cancer with radiological or biopsy confirmed bone metastases, CTx levels in the low-risk range (defined as serum CTx levels in the lowest tertile [<600 ng/L] (Garnero et al. 2003)), and who had received 3–4 weekly anti-bone resorption therapy for a minimum of 3 months were eligible for entry into the REFORM study (Amir et al. 2013). Patients were allocated in an approximate 1:1 fashion to receive pamidronate 90 mg intravenously every 3–4 weeks (control group) or every 12 weeks (de-escalated group) for 48 weeks, stratified according to baseline serum CTx (<400 ng/L and 400-600 ng/L) and duration of prior bisphosphonate use (<6 months and >6 months), using a computer-generated permuted blocks design. As part of the main study, serum CTx and bone-specific alkaline phosphatase (BSAP) levels were measured every 12 weeks for 48 weeks. Self-reported pain was also assessed using the validated Brief Pain Inventory (BPI) and Functional Assessment of Cancer Therapy Bone Pain (FACT-BP) questionnaire at baseline and week 12 on study. Patients allocated to the de-escalated treatment group who remained with telopeptide levels below 600 ng/L continued to receive treatment according to protocol-defined frequency, while patients whose telopeptide levels rose above 600 ng/L remained on study, but thereafter received treatment every 3–4 weeks. All patients received concomitant vitamin D_3_ (800–1000 IU/day) and calcium (1200-1500 mg/day) to prevent hypocalcaemia or secondary hyperparathyroidism. All patients in the main study were asked to participate in this substudy, and only those patients who consented to use of collected specimens for research purposes are included in the present analysis. The study was conducted in accordance with REMARK recommendations for biomarker analyses (McShane et al. 2005), and under institutional ethics approval.

### Biochemical analyses

Serum samples collected after an overnight fast, were allowed to clot for at least 30 minutes then centrifuged at 3,000 rpm for 10 minutes at room temperature. Samples were then processed immediately and stored at −80°C. Serum CTx was measured in a central lab facility with an enzyme-linked immunosorbent assay (ELISA; Beta-Cross Laps/serum assay, Roche Diagnostics Canada Inc, Laval, QC; detection limit 10 ng/L, interassay variability, ~6.7%). Serum BSAP was also measured in a central lab facility by ELISA (Metra Biosystems, San Diego, CA; detection limit 0.7 IU/L, interassay variability ~5.2%). Experimental determination of serum levels of TGF-β (Quantikine, R&D Systems, Minneapolis MN, detection limit 20 pg/ml, interassay variability ~8.3%), activinA (Quantikine, R&D Systems, Minneapolis MN, detection limit 4 pg/ml, interassay variability ~5.9%), P1NP (USCN Life Science Inc., Wuhan China, detection limit 15 pg/ml, interassay variability ~12%), and BSP (Cusabio Biotech Co., Wuhan China, detection limit 2 ng/ml, interassay variability ~12%) were also experimentally measured by ELISA. Urine NTx levels were measured using the Osteomark assay system (Alere, Scarborough ME, detection limit 2nM BCE/mM creatine, interassay variability ~6.9%). All samples were measured in duplicate and concentrations determined following interpolation of standard curves generated from known quantities of recombinant protein. When values were below the threshold of detection for each respective assay, concentrations were assigned as 0.1 below the sensitivity threshold for purposes of statistical analysis.

### Statistical analysis

Descriptive statistics were used to summarize patient characteristics and laboratory measurements across time points. Spearman correlation coefficients were used to investigate for the potential of intra-biomarker variability and to examine potential relationships between different biomarkers at each time point. Univariable logistic regression analysis was conducted to determine the association between selected biomarkers and clinical characteristics and SREs. Multivariable analysis was not conducted due to the small sample size. The nonparametric Mann–Whitney *U* test was used to test for differences in biomarkers at different time points between cohorts. Statistical significance was defined as a p-value of 0.05 or less, and all tests were two-sided. As this study is exploratory no adjustment for multiple testing was made and validation of results from additional studies are required.

### Ethical standards

This study was performed in accordance with ethical standards approved by the Ottawa Health Science Network Research Ethics Board (20120403-01H) and the University Health Network (08-0513-C).

## Results

### Patient cohorts

**Figure 1:**
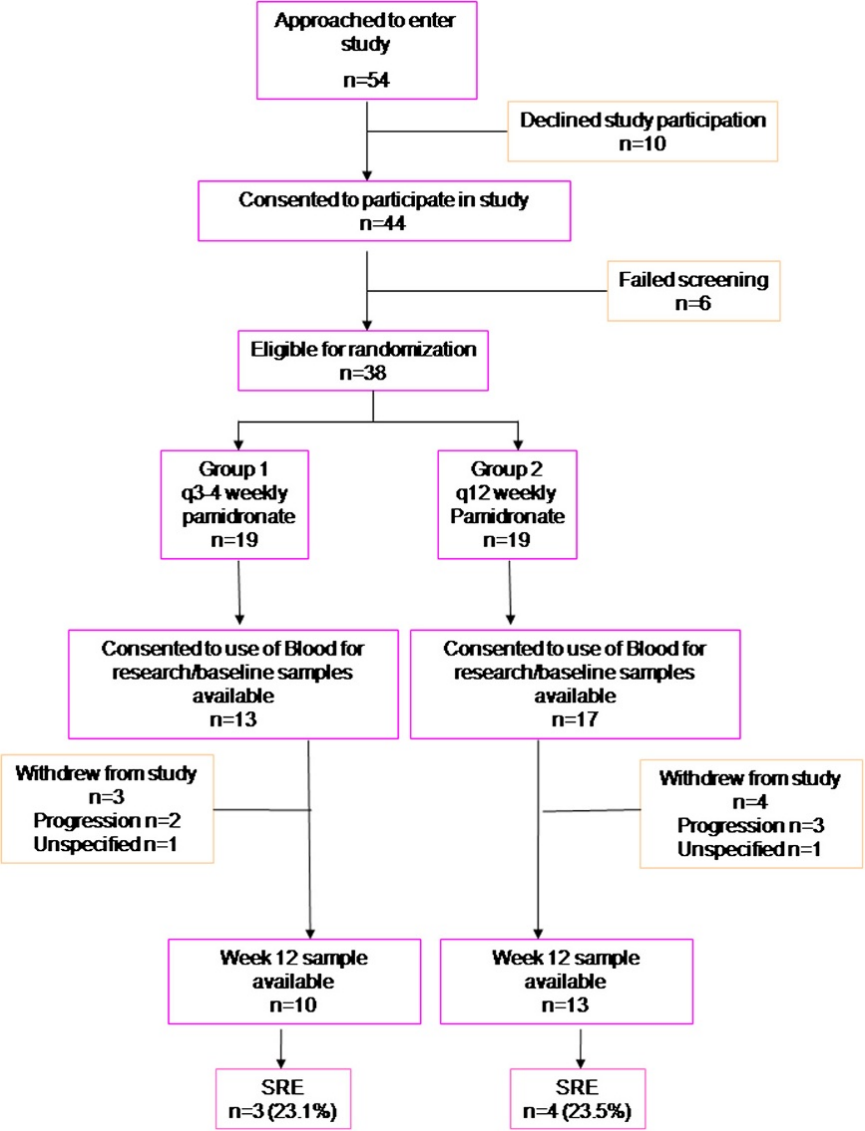
**Consort diagram for the REFORM biomarker substudy.**

Thirty of the 38 patients (79%) who were randomized to the original study provided consent for this substudy (see Figure 1). Of these 30 patients, 13 were randomized to Group 1 (q3-4 weekly) and 17 to Group 2 (12 weekly). Samples were available at week 12 post study entry for 10 patients from Group 1 and 13 from Group 2. Patients were followed for 2 years (one year on study and one additional year of follow up for which frequency of pamidronate use was not specified) during which time seven SREs were observed among the 25 patients with complete data; 3 patients in the control group and 4 patients in the experimental group.

### Biomarker correlations

As each biomarker was measured in duplicate, the intra-biomarker variability was evaluated. The Spearman correlation coefficient exceeded ρ = 0.92 between duplicated measurements for each biomarker at each time point, indicating little to no intra-biomarker variability. Thus, the addition of the duplicate measure added little to no additional information, and as a result, only the first biomarker value is discussed for all further analyses.

The association between different biomarkers at baseline and at week 12, the association of baseline and week 12 biomarker values, as well as the association of the change from baseline to week 12 between different biomarkers, are presented in Additional files 1 and 2: Tables S1 (baseline) and S2 (week 12) respectively. Generally, only weak to moderate correlations were observed between the different biomarker measures. Baseline CTx and BSAP were strongly associated with week 12 measures, while baseline NTx, P1NP, TGF-β and activin-A were all moderately associated for their values at week 12.

Biomarker values at baseline and at week 12 were generally similar between the two treatment arms (Table 1). As can be seen in Table 2, those patients with higher levels of baseline CTx (p = 0.002), BSAP (p = 0.005), P1NP (p = 0.002) and ActivinA (p < 0.001) were significantly more likely to not complete week 12 of the study. It is noted that CTx > 600 ng/L was a trial mandated reason for coming off study early.

**Table 1:** **Biomarker and clinical characteristics**

		Baseline			Week 12		
		All patients	Group 1	Group 2	All patients	Treatment group 1	Treatment group 2
**N**		30	13	17	23	10	13
**CTx ng/L**	Median (IQR)	191 (104, 387)	142 (117, 287)	218 (88, 387)	223 (106, 381)	155 (106, 225)	308 (179, 423)
**BSAP IU/L**	Median (IQR)	18 (14, 30)	16 (14, 30)	18 (14, 29)	17 (14, 29)	16 (12, 29)	18 (16, 25)
**TGF**-**β, ng/ml**	Median (IQR)	22.5 (17.1, 26.3)	24.8 (20.7, 26.3)	20.3 (17.1, 25.5)	21.5 (15.8, 26.6)	21.7 (15.0, 26.6)	21.5 (18.0, 25.7)
**Activin-** **A, pg/ml**	Median (IQR)	597 (419, 1528)	795 (517, 1164)	519 (404, 1666)	560 (483, 1045)	616 (483, 1153)	560 (526, 755)
**NTx 2nM BCE/mM creatine**	Median (IQR)	158 (73, 282)	159 (60, 298)	149 (87, 268)	258 (121, 338)	299 (94, 338)	230 (137, 342)
**P1NP ng/ml**	Median (IQR)	56 (48, 91)	70 (48, 96)	55 (4, 80)	54 (38, 75)	55 (36, 75)	54 (52, 60)
**BSP ng/ml**	Median (IQR)	34 (13, 60)	39 (10, 73)	32 (16, 55)	17 (5, 21)	18 (16, 21)	14 (5, 36)
**FACT**-**BP**	Median (IQR)	13 (5, 25)	8 (5, 14)	16 (11, 26)	13.5 (8, 18)	11 (8, 17)	14 (11, 18)
**BPI**	Median (IQR)	16 (0, 32)	4 (0, 29)	19 (6, 57)	14.5 (2, 21)	13 (1, 20)	15 (7, 21)
**# of SRE** **(%),** **on or post study** **(n** **= 25)**	0	11 (44.0)	5 (45.5)	6 (42.9)	18 (72.0)	8 (72.7)	10 (71.4)
	1	9 (36.0)	3 (27.7)	6 (42.9)	3 (12.0)	2 (18.2)	1 (7.1)
	2	5 (20.0)	3 (27.7)	2 (14.3)	3 (12.0)	1 (9.1)	2 (14.3)
	3				1 (4.0)	0 (0.0)	1 (7.1)

**Table 2:** **Factors associated with failure to complete 12 weeks on study**

		Did not complete week 12	Completed week 12	p-value
**N**		7	23	
**CTx**	Median (IQR)	656 (492, 811)	141 (88, 229)	0.002
**BSAP**	Median (IQR)	44 (26, 68)	15 (14, 22)	0.005
**TGF**-**β**, **ng**/**ml**	Median (IQR)	26.3 (16.8, 30.7)	20.7 (17.1, 25.5)	0.38
**Activin**-**A**, **pg**/**ml**	Median (IQR)	2922 (1666, 9576)	517 (404, 775)	<0.001
**NTx**	Median (IQR)	NA	158 (73, 282)	-
**P1NP**	Median (IQR)	123075 (80014, 178930)	54334 (36692, 77623)	0.002
**BSP**	Median (IQR)	63.9 (55.3, 90.4)	15.9 (8.2, 32.9)	0.004
**FACT**-**BP**	Median (IQR)	15 (7, 36)	12 (5, 23)	0.37
**BPI**	Median (IQR)	57 (12, 65)	6 (0, 29)	0.049
**# of SRE,** **pre** **, study** **(n** **= 25)**	0	1	10	
	1	0	9	
	2	1	4	
**# of SRE** **, on or post,** **study** **(n** **= 25)**	0	2	16	
	1	0	3	
	2	0	3	
	3	0	1	
**Duration of bone mets**, **in months**	Median (IQR)	28 (18, 41)	18 (12, 29)	0.19
**Time from primary to bone mets** (**n** = **25**)	<2 years	1	12	
	2–5 years	0	5	
	>5 years	1	6	

The change in biomarker levels from baseline to week 12 on study was assessed, given that week 12 levels are often used in clinical trials as a surrogate marker of bone metastasis control and hence bisphosphonate efficacy (Lipton et al. 2008; Coleman et al. 2005; Brown et al. 2003; Lipton et al. 1998). Significant increases in biomarker levels from baseline to week 12 for CTx (p < 0.001), BSAP (p = 0.011) and activinA (p = 0.001) were noted for the entire cohort of patients i.e. whether or not they were randomized to de-escalated therapy (Table 3). Comparing between treatment arms, patients in treatment group 2 (q12 weekly arm) had statistically significantly greater increases in CTx (median of 131 versus 17, p = 0.034) and in BSAP (median = 3 versus 0, p = 0.010).

**Table 3:** **Changes in biomarker levels from baseline to 12**-**weeks on treatment**

		Difference all patients	p-value	Difference treatment group 1	Difference treatment group 2	p-value
**N**		23		10	13	
**CTx ng/L**	Median (IQR)	83 (2, 155)	<0.001	17 (−27, 83)	131 (79, 171)	0.034
**BSAP IU/L**	Median (IQR)	1 (0, 4)	0.011	0 (−1, 1)	3 (2, 5)	0.010
**TGF**-**β, ng/ml**	Median (IQR)	0.8 (−2.1, 4.9)	0.28	−0.1 (−2.1, 2.2)	2.4 (−0.0, 4.9)	0.41
**Activin**-**A, pg/ml**	Median (IQR)	111 (28, 526)	0.001	96 (−29, 596)	122 (34, 256)	0.60
**NTx 2nM BCE/mM creatine**	Median (IQR)	42 (−48, 168)	0.15	42 (−15, 179)	45 (−76, 108)	0.54
**P1NP ng/ml**	Median (IQR)	5742 (−6694, 19129)	0.24	1376 (−6694, 8498)	16539 (−1266, 21993)	0.26
**BSP ng/ml**	Median (IQR)	5.8 (−8.0, 9.5)	0.63	0.5 (−15.4, 6.4)	9.8 (0.8, 47.2)	0.14
**FACT** **-BP**	Median (IQR)	0 (−4, 3)	0.99	1 (0, 4)	−2 (−4, 1)	0.053
**BPI**	Median (IQR)	0 (−3, 4)	0.94	2 (0, 7)	−1 (−8, 1)	0.050

### Association between biomarkers and pain

Baseline pain scores tended to be higher in patients randomized to 12-weekly therapy (p = 0.051 for FACT-BP and p = 0.12 for BPI), but pain scores were similar at the week 12 time point (p = 0.64 for FACT-BP and p = 0.55 for BPI). Interestingly, pain scores as measured by both FACT-BP (p = 0.053) and BPI (p = 0.050) tended to be more reduced from baseline to 12 weeks in the patients treated with the de-escalated regimen as compared to 3–4 weekly treated patients (Table 3), although it is noted that the p-value was low and did not attain the pre-defined level of statistical significance.

### Association between biomarkers and SRE incidence

Results from univariable exploratory analyses to determine whether circulating levels of biomarkers were significantly different between patients who experienced on study SREs versus those that did not are shown in Table 4. Interestingly, 6/14 (43%) patients who had previous SREs prior to study entry, had an on study SRE, while only 1/11 (9%) without a prior SRE experienced an on study SRE (p-value = 0.088). At 2 years of follow up, SRE rates remained similar between the two cohorts, with 3/11 (27%) of 3–4 weekly treated patients and 4/14 (29%) of 12 weekly treated patients having experienced SREs (p-value = 0.94).

**Table 4:** **Association of biomarkers with odds of having an on or post**-**study SRE**

		Baseline		Change from Baseline to 12 Weeks	
		Odds ratio (95% CI)	p-value	Odds ratio (95% CI)	p-value
**CTx**	/10 unit change	1.00 (0.94, 1.07)	0.99	1.04 (0.95, 1.14)	0.39
**BSAP**	/unit change	1.03 (0.97, 1.09)	0.36	0.90 (0.73, 1.11)	0.32
**TGF**-**β**	/unit change	1.05 (0.90, 1.23)	0.54	0.98 (0.83, 1.16)	0.83
**Activin**-**A**	/100 unit change	0.98 (0.89, 1.07)	0.60	0.99 (0.93, 1.04)	0.64
**NTx**	/10 unit change	1.00 (0.97, 1.02)	0.69	1.01 (0.97, 1.06)	0.56
**P1NP**	/10000 unit change	0.97 (0.79, 1.18)	0.75	0.98 (0.80, 1.20)	0.84
**BSP**	/10 unit change	1.00 (0.63, 1.60)	0.99	0.94 (0.60, 1.47)	0.79
**FACT**-**BP**	/unit change	1.13 (1.01, 1.28)	0.041	0.99 (0.87, 1.14)	0.93
**BPI**	/unit change	1.03 (0.99, 1.08)	0.16	1.00 (0.94, 1.06)	0.93
**Duration of bone metastases**	/month	1.03 (0.99, 1.08)	0.14		
**Pre**-**Study SRE**	Yes versus no	7.50 (0.74, 75.72)	0.088		
**Treatment Group**	2 versus 1	1.07 (0.18, 6.21)	0.94		

No significant association was observed for any of the baseline biomarkers, including the more commonly used markers such as CTx and NTx (Table 4), as prognostic factors for experiencing a SRE. Similarly, the change in level from baseline to week 12 was not significantly associated with experiencing on study SREs for any biomarker (Table 4). Of all the parameters measured, only baseline pain as measured by FACT-BP was statistically significantly associated with development of an on or post-study SRE (p = 0.041, Table 4). Changes in pain levels from baseline to 12 weeks, as measured by FACT-BP, were not associated with SRE development (Table 4).

## Discussion

De-escalated bisphosphonate therapy is being increasingly utilized after data from the large randomized ZOOM and OPTIMIZE-2 trials showed that skeletal outcomes are similar to those of patients receiving standard 3–4 weekly treatment (Amadori et al. 2013; Hortobagyi et al. 2014). As confirmed in our present study, a common feature of studies of de-escalated bisphosphonate therapy appears to be modest increases in telopeptide levels from baseline to week 12 in the de-escalated arm, while levels are maintained in the standard frequency arm. Despite this, no differences in either the number of SREs (REFORM (Amir et al. 2013) and current study), time to first on-study SRE (ZOOM (Amadori et al. 2013)) or SRE rate (OPTIMIZE-2 (Hortobagyi et al. 2014)) were observed between the two treatment groups. This raises the question of whether or not circulating telopeptide levels on treatment are truly related with SRE risk. With the continuing trend of clinical studies being designed to use measurement of these markers as endpoints to determine drug efficacy, and the fact that patients on these drugs experience SREs regardless of the level of telopeptides, it is of the utmost importance to gain a better understanding of the relationship between biomarker measures and subsequent SRE risk.

In the current analysis a range of recognized bone-turnover or metastasis biomarkers were measured. Urinary N-telopeptide (uNTx) is a fragment of collagen I produced during osteolysis of the bone and has frequently been used to monitor bone resorption in both osteoporotic (Yoshimura et al. 2011; Garnero 2008; Reginster et al. 2001) and bone metastases (Lipton et al. 2008; Lipton et al. 2011; Coleman et al. 2005) patients. Elevated levels of uNTx have been shown to correlate with bone turnover (Rosen et al. 1994; Gorai et al. 1997; Clemens et al. 1997). P1NP is a marker of bone formation (Garnero et al. 2000b) which has been shown to correlate with presence of bone metastases in prostate cancer patients (Garnero et al. 2000a), and previously was shown to be associated with increased bone fractures (Chevrel et al. 2007). TGF-β plays a critical role in the exacerbation of osteolytic metastatic disease by contributing to continued osteoclast activity and aggressive and invasive tumour phenotypes (Ivanovic et al. 2003). Furthermore, plasma TGF-β levels have been found to correlate with disease stage and bone metastasis in breast cancer (Baselga et al. 2008; Desruisseau et al. 2006). Similarly recent studies have suggested that the TGF-β family member, activinA, also plays a role in metastatic and osteoclastic processes (Leto et al. 2006; Risbridger et al. 2001; Fuller et al. 2000). Plasma levels of activinA were shown to be higher in breast cancer patients with bone metastases as compared to those without and to positively correlate with the number of bone metastases (Reis et al. 2002). Lastly, tumour-derived expression of BSP also correlates with subsequent development of osseous metastases, as it has been shown that baseline serum BSP levels were elevated in ~90% of women who went on to develop bone metastases, while not elevated in patients who developed visceral metastases (Diel et al. 1999).

None of the measured parameters at baseline nor their changes from baseline to week 12, were observed to predict for subsequent SREs. The fact that other more routinely used markers such as CTx and uNTx were not significantly different in patients who experienced SRE versus those that did not in our study may simply be a result of the fact that these cohorts were too small to discern those differences. Although previous studies have suggested that median levels of many bone turnover biomarkers tend to be higher in the patients who experienced SREs (Coleman et al. 2008), additional studies lead us to question this notion in bone metastatic patients considered to be low risk due to bone turnover markers closer to the ‘normal range’. Recent results with NTx have suggested that in patients with uNTx levels considered to be in the low range, >90% of the patients who experienced SREs had no documented increase in NTx prior to their SRE (Lipton et al. 2013). A further possibility is that in patients with low baseline levels of telopeptides changes in these markers are not predictive of bone outcomes. It remains unclear if this observation can be extrapolated to patients with higher baseline biomarker levels.

Of all the parameters measured in the current study, it is interesting that pain at baseline was prognostic for occurrence of subsequent SREs. Previous analyses in metastatic breast cancer patients treated with the bisphosphonate zoledronic acid, also found that baseline pain was associated with time to first SRE in univariable analysis (Brown et al. 2010). However, in this study, pain was not associated with skeletal fractures, but was significantly associated with need for radiation treatment. Of the 7 patients who experienced SREs in REFORM all required radiotherapy (although each may have had additional SREs of other types in conjunction), which may explain the observed association between baseline pain and occurrence of SREs. Despite this, given that pain significantly affects a patient’s quality of life and is associated with need for radiation treatment for skeletal complications, its measurement in future studies is warranted.

With data suggesting that de-escalation of bisphosphonate therapy may be equally therapeutically effective yet also spare patients exposure to the detrimental effects of excessive cumulative dose of bisphosphonates (Amadori et al. 2013; Amir et al. 2012), it is essential to better understand the pharmacodynamics of bone turnover markers in response to these agents if clinical decisions are to be made based on these factors. While markers such as NTx and CTx may be very effective as prognostic indicators of bone turnover in patients with bone metastases starting bone targeted therapies, additional markers assessing risk of SRE may be required. This may be particularly important in those cases where patients are found to have NTx and CTx levels considered to be in the “low” or “normal” range with bone-targeted treatments. Furthermore, it is unclear what effects concomitant anti-cancer therapy may have on the relative levels of these markers that may further confound their utility as predictive markers on treatment. Recent studies have shown that use of exemestane results in increased levels from baseline for BSAP, P1NP and CTx, while addition of everolimus to exemestane resulted in decreased levels of the same biomarkers after baseline (Gnant et al. 2013). Importantly, the changes in biomarker levels were observed in patients irrespective of the presence of bone metastases, highlighting the effect of chemotherapy on normal bone homeostasis in general. Interestingly, a large recent biomarker driven study in patients starting fulvestrant also showed significant decreases in uNTx from baseline to on treatment (Clemons et al. 2014). As these patients had no change in their bone-targeted therapy at the time of study entry, this confirms that changes in anticancer therapies can alter bone biomarker levels.

This study has a number of limitations. It was specifically designed as a small study to assess the feasibility of randomizing patients to de-escalated therapy. As such it has a small cohort and even with the extended follow up, a low number of SREs has occurred. Furthermore, 5 patients were lost to follow up over the year following the end of the REFORM study, further restricting our sample size. It is also of interest to note that only pain as assessed by FACT-BP but not by BPI was associated with SRE occurrence, despite both tests being validated measurement tools. The reasons for this remain unclear. Finally, all patients enrolled in our study had low baseline levels of CTx and therefore, our results may not be generalizable to patients with bone metastases who present with a greater magnitude of bone resorption.

## Conclusions

In conclusion, these findings show that no significant changes in any of the biomarkers tested were observed between the standard and the de-escalated treatment groups. Exploratory analysis suggested that biomarkers of bone activity do not appear to predict for SREs in the ‘low risk’ bone metastases patients. Bone pain does however appear to be associated with SRE occurrence, a finding which warrants evaluation in larger studies.

## Electronic supplementary material

Additional file 1: Table S1: Spearman Correlation of Baseline Biomarkers. (DOC 30 KB)

Additional file 2: Table S2: Spearman Correlation of Change in Biomarkers from Baseline to Week 12. (DOC 30 KB)

## Abbreviations

BPI: Brief pain index
BSAP: Bone specific alkaline phosphatase
BSP: Bone sialoprotein
CTx: C-telopeptide
FACT-BP: Functional assessment of cancer therapy-bone pain
TGF-β: Transforming growth factor beta
NTx: N-telopeptide
P1NP: Pro-collagen type 1 amino terminal peptide
SRE: Skeletal related event.
