# Supplementary material for: Effects of de-escalated bisphosphonate therapy on bone turnover biomarkers in breast cancer patients with bone metastases
Source: Springerplus. 2014 Oct 1;3:577. doi: 10.1186/2193-1801-3-577 (PMC4194305; doi:10.1186/2193-1801-3-577)
Supplement: Supplementary file 1 — Additional file 1: Table S1: Spearman Correlation of Baseline Biomarkers. (DOC 30 KB) [file 40064_2014_1283_MOESM1_ESM.doc]

**Additional file 1: Table S**1. Spearman Correlation of Baseline Biomarkers

|  | **BAP** | **TGF** | **ACT-a** | **NTx** | **P1NP** | **BSP** | **FACT-BP** | **BPI** | **Baseline with Week 12 value** |
| --- | --- | --- | --- | --- | --- | --- | --- | --- | --- |
| **CTx** | 0.69 | 0.21 | 0.64 | 0.49 | 0.36 | 0.49 | 0.32 | 0.45 | 0.86 |
| **BSAP** |  | 0.06 | 0.79 | 0.42 | 0.45 | 0.36 | 0.20 | 0.24 | 0.90 |
| **TGF-β, ng/ml** |  |  | 0.22 | 0.42 | 0.55 | 0.27 | 0.23 | 0.26 | 0.68 |
| **Activin-A, pg/ml** |  |  |  | 0.51 | 0.64 | 0.58 | 0.07 | 0.16 | 0.71 |
| **NTx** |  |  |  |  | 0.23 | -0.29 | 0.06 | -0.25 | 0.48 |
| **P1NP** |  |  |  |  |  | 0.60 | 0.25 | 0.42 | 0.62 |
| **BSP** |  |  |  |  |  |  | -0.07 | 0.28 | 0.28 |
