# Supplementary material for: Effects of de-escalated bisphosphonate therapy on bone turnover biomarkers in breast cancer patients with bone metastases
Source: Springerplus. 2014 Oct 1;3:577. doi: 10.1186/2193-1801-3-577 (PMC4194305; doi:10.1186/2193-1801-3-577)
Supplement: Supplementary file 2 — Additional file 2: Table S2: Spearman Correlation of Change in Biomarkers from Baseline to Week 12. (DOC 30 KB) [file 40064_2014_1283_MOESM2_ESM.doc]

**Additional file 2: Table S**2. Spearman Correlation of Change in Biomarkers from Baseline to Week 12.

|  | **BAP** | **TGF** | **ACT-a** | **NTx** | **P1NP** | **BSP** | **FACT-BP** | **BPI** |
| --- | --- | --- | --- | --- | --- | --- | --- | --- |
| **CTx** | 0.23 | 0.36 | 0.39 | -0.01 | 0.11 | 0.54 | -0.16 | -0.21 |
| **BSAP** |  | 0.17 | 0.08 | -0.20 | 0.30 | 0.37 | -0.26 | -0.33 |
| **TGF-β, ng/ml** |  |  | 0.09 | -0.19 | 0.45 | 0.22 | 0.09 | 0.11 |
| **Activin-A, pg/ml** |  |  |  | 0.05 | 0.38 | 0.68 | 0.02 | 0.37 |
| **NTx** |  |  |  |  | -0.32 | -0.27 | 0.18 | 0.18 |
| **P1NP** |  |  |  |  |  | 0.78 | -0.00 | 0.23 |
| **BSP** |  |  |  |  |  |  | -0.42 | 0.00 |
